# Supplementary material for: A2AR Expression and Immunosuppressive Environment Independent of KRAS and GNAS Mutations in Pseudomyxoma Peritonei
Source: Biomedicines. 2023 Jul 20;11(7):2049. doi: 10.3390/biomedicines11072049 (PMC10377259; doi:10.3390/biomedicines11072049)
Supplement: Supplementary file 1 [file biomedicines-11-02049-s001.zip › biomedicines-2443595-supplementary.pdf]

Supplementary Materials

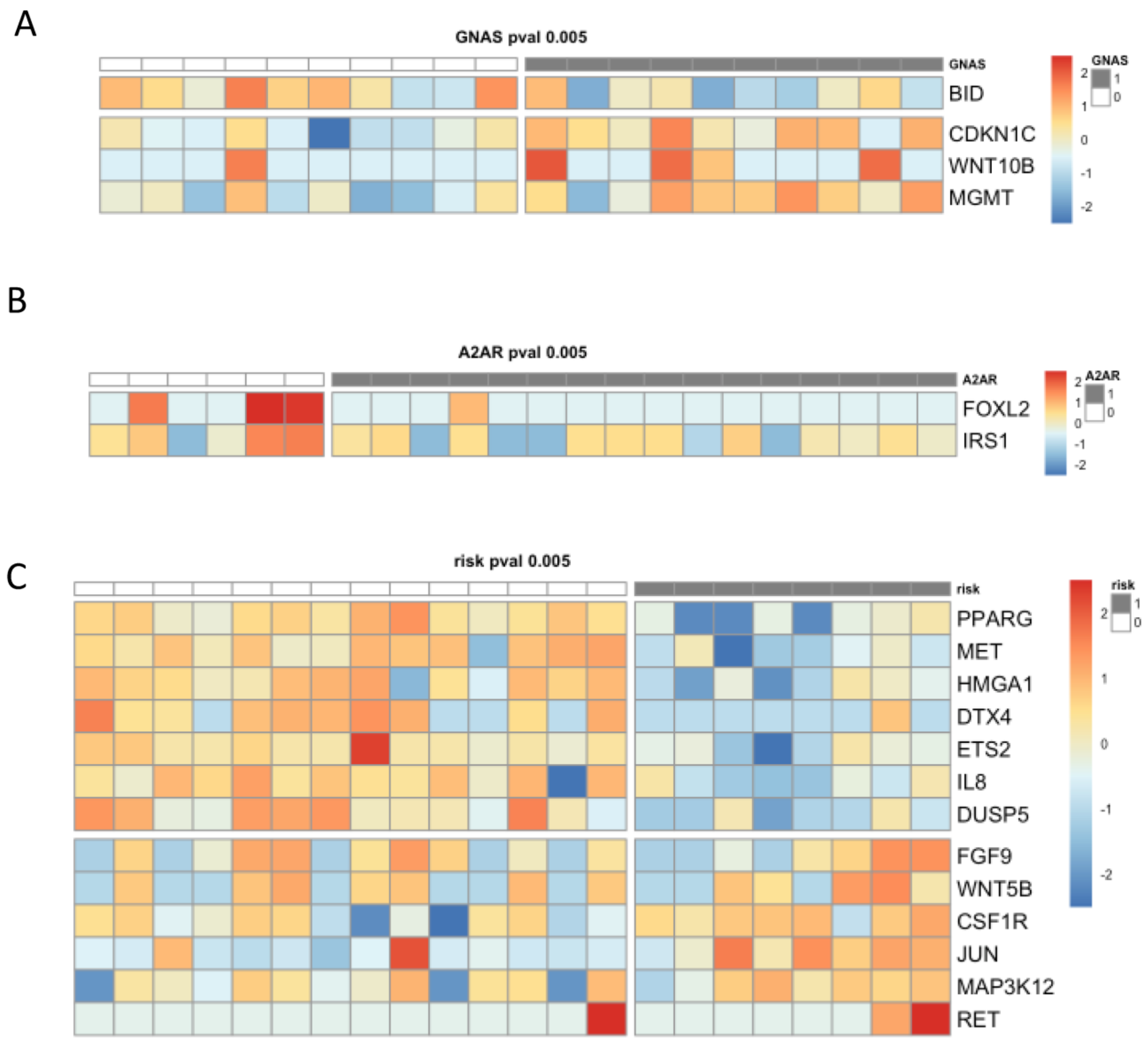

Figure S1. Expression analysis results. (**A**) Compared expression between GNAS mutated and wild type cases. Legend: 0: GNAS wild type; 1: GNAS mutation (**B**) Compared expression between A2AR positive and negative cases. Legend: 0: A2AR positive; 1: A2AR negative. (**C**) Compared expression between high- and low- risk cases. Legend: 0: low risk; 1: high risk.

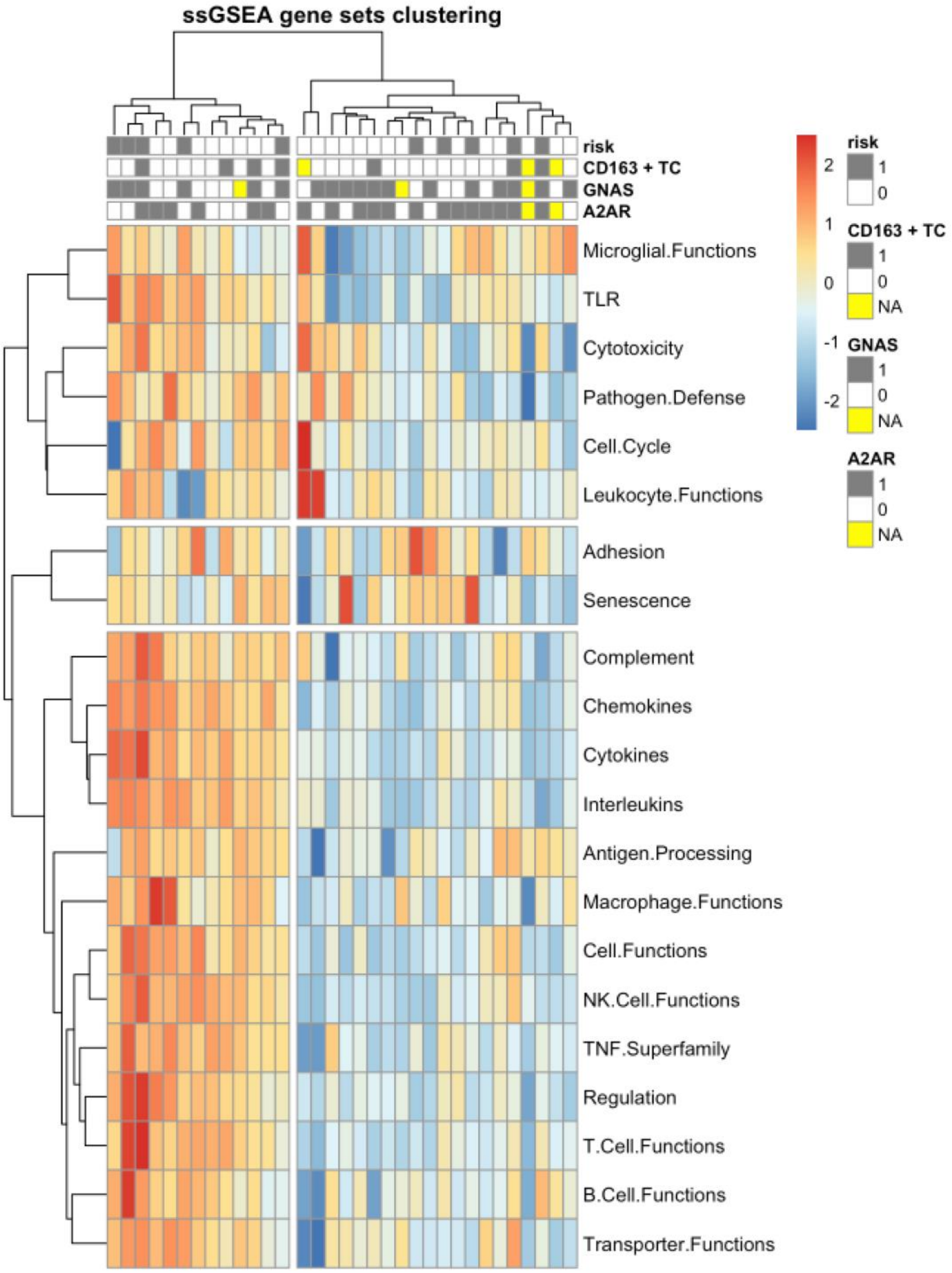

Figure S2. GSEA analysis. A group of 13 patients showed enrichment of genes whose expression is enhanced in immunological and inflammation pathways. Legend: 1: positive; 0: negative; NA: not available; TC: tumor cells.
